# Factors related to delay in initiating post-exposure prophylaxis for rabies prevention among animal bite victims: a cross-sectional study in Northwest of Iran 

**DOI:** 10.30476/beat.2020.85134

**Published:** 2020-10

**Authors:** Ehsan Sarbazi, Mohamadreza Sarbazi, Saber Ghaffari-fam, Towhid Babazadeh, Sohrab Heidari, Khadijeh Aghakarimi, Ismail Jamali, Ali Sherini, Javad Babaie, Ghader Darghahi

**Affiliations:** 1 Tabriz Health Services Management Research Center, Health Management and Safety Promotion Research Institute, Tabriz University of Medical Sciences, Tabriz, Iran; 2 Road Traffic Injury Research Center, Student Research Committee, Tabriz University of Medical Sciences, Tabriz, Iran; 3 PhD student in Applied ELT at Ilam University, Faculty of humanities and foreign languages Ilam, Iran; 4 School of Nursing of Miyandoab, Urmia University of Medical Sciences, Urmia, Iran; 5 Department of Public Health, Sarab Faculty of Medical Sciences, Sarab, Iran; 6 Tabriz Health Center, Tabriz Rabies Prevention and Treatment Center, Iranian Center of Excellence in Health Management, Tabriz University of Medical Sciences, Tabriz, Iran; 7 Department of Health Services Management, Iranian Center of Excellence in Health Management, School of Management and Medical Informatics, Tabriz University of Medical Sciences, Tabriz, Iran; 8 MSc of Epidemiology, Research Center of Psychiatry and Behavioral Sciences, Razi Hospital, Tabriz University of Medical Sciences, Tabriz, Iran

**Keywords:** Post-exposure prophylaxis, Rabies, Animal bite, Iran

## Abstract

**Objectives:**

The aim of the present study was to identify factors associated with h delayed initiation of post-exposure prophylaxis (PEP) among animal bite victims.

**Methods:**

This cross-sectional study assessed biting patterns among 3032 cases that were referred to Tabriz Rabies Center. The delay was described as the initiation of PEP more than 48 hours (h) after possible exposure to the rabies virus. Determinants of delay in initiating PEP were recognized by a decision tree model.

**Results:**

Totally, 8.5% of the victims who were bitten by an animal had a delay of more than 48 h in the initiation of PEP. The relative frequency of a delay more than 48 h in females was higher than in males (12.9% compared to 8.5%) (p-value= 0.004). Relative frequency of a delay of more of 48 h from carnivorous (dog, jackal, fox) was significantly less than others (p-value< 0.001). Of the decision tree, the overall classification accuracy was 89.5%, with 44.1% sensitivity and 92.3% specificity. The identified variables included gender, biting place (rural, urban), and type of animal.

**Conclusion:**

according to the results of the present study, among the various variables that affect the delayed initiation of PEP, rural residents and being female, in particular, were the major factors associated with a delay in the initiation of PEP for rabies prevention. We found relatively low rates of vaccine completion. Our findings indicat that providing training and patient education are required to ensure the completion of appropriate treatment.

## Introduction

Animals attack is still a major health and social issue worldwide. An animal bite is the main source of transmission of rabies to humans, which has not yet been controlled in most parts of the world (1). Approximately, 85 to 90% of animal-bite injuries inflicted upon human beings are caused by dogs, 5 to 10% by cats and 2 to 3% by humans and rodents (2). In low-income countries, several studies have demonstrated that dogs account for 76 to 94% of animal-bite injuries resulting in a high prevalence of rabies and higher fatality rates due to poor access to anti-rabies post-exposure treatment (3). Rabies is an infectious viral disease that leads to death after initiating clinical symptoms. In up to 99% of the cases, domestic dogs are responsible for rabies virus transmission to humans. However, rabies can affect both domestic and wild animals (4). It spreads to people through bites or scratches, and usually via saliva (5).

After the onset of clinical symptoms, there is no effective treatment for rabies. Therefore, the currently recommended intervention strategy is to remove and neutralize the infectious virus before it enters the nervous system (6). Inappropriate or delayed treatment will result in an increased risk of acute infection and re-infections. Therefore, post-exposure prophylaxis (PEP) is necessary to reduce the risk of infection. In the case of potential rabies exposure, the World Health Organization (WHO) recommends immediate wound washing, administration of anti-rabies vaccine and infiltration of purified rabies immunoglobulin inside and around the wound for severe categories of exposure (7).

Annually, 180000 animal bite cases are reported in Iran, all of whom receive PEP because all biting animals such as cats, dogs, and wolf are considered to be reservoirs of rabies (8). Moreover, 69.4% out of 81% of people who are bitten by dogs are related to domestic dogs (9).

In this regard, the community needs to be adequately educated to ensure them to take care of themselves in preventing rabies and the consequences caused by animal bites (10). Besides, by proper education of to dog keepers, proper measures should be taken as for vaccination of the dogs. Individuals should be trained in such a way to refer to health centers for immediate and necessary post-exposure prevention interventions (11). The time interval between animal bites and initiation of PEP has been seen to vary from hours to weeks (12). Timely completion of vaccination is essential both in local infections and in the probability of transmission of rabies to humans. Rabies vaccination is free of charge 24/7 for all victims of animal bites in Iran (8, 13).

Treating a rabies exposure, where the average cost of rabies PEP is US$ 40 in Africa, and US$ 49 in Asia, can be a catastrophic financial burden on affected families whose average daily income is around US$ 1–2 per person (14). PEP is a substantial health investment that reduces the burden of rabies and improves community health (15). Surveillance of animal-related injuries could provide useful information for planning and evaluating public health interventions (16). Accurate data on animal bite incidence may lead to more effective policy-decision making towards more efficient resource allocation to primary health care so as to reduce human and animal rabies cases in the country. Previous studies have shown that factors such as place of residence and distance from the center affect PEP (17). This study aimed to investigate the reasons of delay more than 48 h in initiating the PEP in Tabriz city, Iran.

## Material and Methods

*Study design*

This cross-sectional study investigated the patterns of animal bites among the cases referred to the rabies center of Tabriz between March 1 2013 and February 29 2019. A sequential sampling of 3032 patients with animal bite history visiting the rabies center was performed. The data were taken from rabies surveillance forms used in the district. These forms are used to identify and follow-up the suspected bites. When a bite case is admitted to a health center, a health worker for communicable disease control performs an examination, decides whether prophylaxis is necessary, fills out the form and follows-up the case until the end of PEP. Data mining was used via the decision tree model to identify factors affecting delay in vaccination 48 h subsequent to bites to prevent rabies. 

*Study Site*

Post-prophylactic rabies centers have been established in provinces and even in small towns in Iran under the supervision of the Ministry of Health and Medical Education. The study was conducted at the rabies center of Tabriz which is affiliated to Tabriz University of Medical Sciences.

*Study population*

An animal bite was defined as any animal bites caused by mammals. Data were extracted for the patients who were registered at the health center and referred to the health center of Tabriz city to receive a rabies vaccination. *All cases of animal bite in all age and gender groups which had been referred to the Rabies Center of Tabriz were investigated.*

*Ethics approval and consent to participate*

*The institutional ethical review board reviewed and approved the study protocol in Tabriz, Iran (*IR.TBZMED.REC.1397.1096). All data have been anonymized and treated confidentially.

*Data collection*

We collected data per span (year) on the number of animal bite injuries. To collect the data, we used the following a structured data abstraction tool to extract the required data form recoded forms: age (<10 years old, 10-20 years old, 20-30 years old, 30-40 years old, 40-50 years old, >50 years old); sex (male, female); occupation (clerk, laborer, retired, housekeeper, agriculture, student, driver, self-employed); biting place (urban area, rural area); being stray (yes/ no); place of injury in human body (upper limb of the human body, lower limb of the human body); puncture wounds (yes, no); animal status (live, escaped, dead); time of event (before 7 A.M, 713, 13-19, 19-24). The dependent variable was: PEP initiation delay which was defined as the initiation of PEP more than 48 h after an animal bite. We looked into the time of injury and time to visit with the respondents. In the present study, PEP delay was defined as the initiation of PEP more than 48 h after a possible exposure to the rabies virus. The variable was coded according to: 0= “prompt PEP”, 1= “delay of initiation of PEP”.

*Input variables*

After cleaning and preparation of the data, the final dataset consisted of 3032 records. The outcome of interest was the delay more than 48 h in initiation of PEP which was assessed by the model based on several input variables presented in Table 1.

*Construction of decision tree*

As the number of instances in this study was enough, the decision tree was built with the holdout method in which data are randomly partitioned into two independent sets, a training set and a test set.

We divided the original dataset into two parts using stratified random sampling based on the target variable, with the training dataset containing about 70%, and 30% of the participants as the testing dataset. The Gini impurity index was chosen as the attribute selection measure. Where pi is the probability that an example in D belongs to class Ci, and is estimated by [Ci, D|/|D|. The sum is computed over m classes. The Gini index considers a binary split for each variable. In the decision tree, the first variable (root) is the most important factor and variables far away from the root are the next important factors in classifying the data. For easy understanding, the decision tree can be converted to a set of If-Then rules by tracing the path from the root node to each terminal (leaf) node. All the variables in one path are considered as predictors (If part) and the class label of the leaf node is expected outcome (Then part). These rules are extracted just by top down tracing of the path and there is no rank or weight for the rules. For classifying a new person, we should start with the root node of the decision tree and moving along the path that the person belong it until the leaf node is reached. The decision about the person is determined based on value of leaf node which is usually positive or negative with a certain probability (18).

In addition, missing values for numerical features were handled by setting their values to average value, and replacing the most frequent value for nominal features.

Classification is the process of finding a model (or function) describing and distinguishing data classes on concepts using the model to predict object classes (19). Classification models are based on training data, the independent variables and target variables of which are known, and then they are used for estimating the target variable on a new dataset. There are diverse classification methods: neural networks, decision trees and regression. Decision tree classifies the nominal target variables, it is called a “classification tree”, and when it’s used to forecast a continuous, it is termed a “regression tree”. In the present study, concerning the type of dependent variable, the classification and regression trees (CART) applied to assess the effect of each variable on the probability of delay more than 48 h in the initiation of PEP. In the first step of the CART model, the input data is concentrated at the top of the tree, at the first node. Then this so-called “root node” is divided into two child nodes on the basis of a predictor variable (splitter) that maximizes the homogeneity (i.e., purity) of the two child nodes. This process is continued repeatedly for each child node until all the data in each node have the highest possible homogeneity. This node is called a “leaf” or terminal node, and has no branches.

Where J is the number of classes or the target variables, π(j) is the prior probability for class j, p (j|m) is the probability that node m includes observations of class j, and Gini (m) is the Gini index, which indicates impurity in node m. The Gini index is 0, if all the observations in a single node belong to a unique class that displays the least impurity, and is equal to 1-1/i, if results in different classes in one node are of the same proportion. In this situation, the maximum tree that overfits the training data has been created. Reducing the complexity of the end tree and generating simpler trees, based on a cost-complexity algorithm, the tree will be “pruned”. In the CART method, the decision tree is getting bigger and more so until each terminal node has the same observations. Gini index the degree or probability of a particular variable being wrongly classified when it is randomly chosen.

*Statistical analysis*

Mean ± standard deviation (SD) for continuous variables and frequencies (%) for categorical variables were used to demonstrate baseline characteristics of the participants. Factors associated with PEP treatment with dichotomized and categorical variables were tested using chi-square test as a univariate study.

## Results

In total, 3032 animal bites were recorded through 2013-2019; with no human rabies cases. The mean age of subjects was 33.71± 18.50 years. Cases ranged in age from 1 to 91 years, and 2438 (80.4%) were males. Demographic and injury Characteristics of the study population, and the number of victims bitten by the animals are shown in Table1. Majority of the animal bite victims in this study (47.5%) were self-employed. In sum, 259 (8.5%) of the victims in the study course had a delay of more than 48 h of rabies vaccination. Most of the exposures were reported from health centers in urban areas 2094 (69.1%). In 1793 (59.2%) of the cases, the animal involved in the bite was a carnivorous (dog, jackal, fox). The most bitten body sites were upper limb of the human body (66.2%) and lower limb of the human body (33.8%).

According to the results of the chi-square test, the relative frequency of a delay of more than 48 h in females was higher than the males (12.9% vs. 8.5%) (p-value= 0.004). Relative frequency of a delay of more of 48 h from carnivorous (dog, jackal, fox) was significantly less than others (p-value< 0.001). There was a significant difference between the animal status and delay in initiation of PEP according to the chi-square test (p-value< 0.001) to be more specific the relative frequency of delay was higher in cases that the biter animal is dead after bite.

Table 1 shows the results of univariate analysis. First, based on univariate analysis for data mining, all variables having an important association (p-value< 0.1) with the more than 48 h delay in PEP vaccination were entered into the decision tree model. Gender, type of animal, being stray, animal status, and age groups were selected for analysis in the decision tree. Then, based on the CART algorithm, the depth of the tree was determined to be equal to three.

**Fig. 1:**
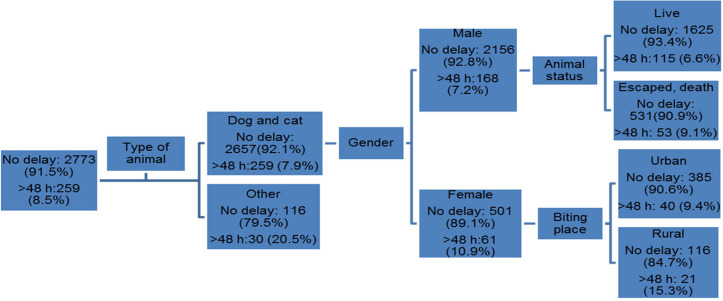
Decision tree model for predicting delay of more than 48 h in the initiation of PEP

The Gini index, as an impurity function of the CART algorithm, showed that the most important variables for predicting the delay of PEP include: type of animal, gender, animal status and biting place, respectively [Figure 1].

We developed a decision tree with instruction set (3032 records). The criteria for building the tree included minimum record number per node, the pruning process and attribute selection measures; minimum number represents a stopping condition for further data partitioning at decision nodes. Briefly, various decision trees with different ‘minimum records’ were built and the value of 50 was chosen which resulted in best performance. The Gini index was selected as measure of attribute selection, and the tree were kept unpruned. The overall classification accuracy was 89.5%, with 44.1% sensitivity and 92.3% specificity.

**Table 1:** Univariate analysis of factors affecting in delay in initiation PEP

**p-value **	**Relative frequency percentage of delay mare than 48 h**	**Delay more than 48 h**		**Total**	**Subgroups **	**Factors affecting the delay**
		**Yes, N (%)**	**No, N (%)**			
0.004	8.5	191 (73.7)	2247 (81.0)	2438 (80.4)	Male	**Sex**
	12.9	68 (26.3)	526 (19.0)	594 (19.6)	Female	
0.246	12.5	24 (9.3)	191 (6.9)	215 (7.1)	Clerk	**Occupation **
	5.9	11 (4.2)	185 (6.7)	196 (6.5)	Laborer	
	15.2	11 (4.2)	72 (2.6)	83 (2.7)	Retired	
	11.7	34 (13.1)	289 (10.4)	323 (10.7)	Housekeeper	
	8.4	10 (3.9)	119 (4.3)	129 (4.3)	Agriculture	
	8.8	43 (16.6)	487 (17.6)	530 (17.5)	Student	
	7.4	8 (3.1)	107 (3.9)	115 (3.8)	Driver	
	8.9	118 (45.6)	1322 (47.7)	1440 (47.5)	Self-employed	
0.516	11.1	3 (1.2)	27 (1.0)	30 (1.0)	Before 7 A.M	**Time of event **
	9.4	215 (83.0)	2265 (81.7)	2480 (81.8)	713	
	10.3	27 (10.4)	262 (9.4)	289 (9.5)	1319	
	6.3	14 (5.4)	219 (7.9)	233 (7.7)	19-24	
0.056	8.6	167 (64.5)	1927 (69.5)	2094 (69.1)	Urban area	**Biting place **
	10.8	92 (35.5)	846 (30.5)	938 (30.9)	Rural area	
0.001	7.5	126 (48.6)	1667 (60.1)	1793 (59.2)	Carnivorous (Dog, Jackal, Fox)	**Type of animal **
	10.4	103 (39.8)	989 (35.7)	1092 (36.0)	Cat	
	25.8	30 (11.6)	116 (4.2)	146 (4.8)	Other	
0.08	10.7	110 (42.5)	1020 (36.8)	1130 (37.3)	Yes	**Being stray **
	8.4	149 (57.5)	1753 (63.2)	1902 (62.7)	No	
0.397	9.1	169 (65.3)	1837 (66.2)	2006 (66.2)	Upper limb of the human body	**Place of injury in human body **
	9.6	90 (34.7)	936 (33.8)	1026 (33.8)	Lower limb of the human body	
0.163	9.59	231 (89.2)	2408 (86.8)	2639 (87.0)	Yes	**Entering saliva of animal into human body **
	7.6	28 (10.8)	365 (13.2)	393 (13.0)	No	
0.112	9.5	242 (93.4)	2524 (91.0)	2766 (91.2)	No	**Puncture of wounds **
	6.8	17 (6.6)	249 (9.0)	266 (8.8)	Yes	
0.001	8.4	175 (67.6)	2075 (74.8)	2250 (74.2)	Live	**Animal status**
	11.2	75 (29.0)	667 (24.1)	742 (24.4)	Escaped	
	29.0	9 (3.5)	31 (1.1)	40 (1.4)	Dead	
0.191	11.3	39 (15.1)	343 (12.4)	382 (12.6)	<10	**Age groups **
	9.7	30 (11.6)	309 (11.1)	339 (11.2)	10-20	
	7.1	47 (18.1)	659 (23.8)	706 (23.3)	20-30	
	11.4	55 (21.2)	482 (17.4)	599 (19.8)	30-40	
	8.0	35 (13.5)	436 (15.7)	409 (13.5)	40-50	
	9.7	53 (20.5)	544 (19.6)	597 (19.7)	>50	

According to figure 1, four rules of this structure can be understood:

1. If the biting animal is other animals, 20.5% will have a delay of more than 48 h.
2. If the biter animal is a dog or cat and gender of the victim is female, 10.9% had a delay of more than 48 h.
3. If the biting animal is a cat or dog and the gender of the victim is female, as well as biting place is rural area, 15.3% will have a delay of more than 48 h.
4. If the biting animal is a cat or dog and gender of the victim is male, as well as animal status is escaped or dead, 9.1% will have a delay of more than 48 h.

## Discussion

Post-exposure vaccination against rabies is essential for prevention of this fatal disease. Obviously, several essential factors in the proper implementation of PEP, are available. Many factors influence timely access to PEP and its administration. This study investigated the factors causing delay in receiving anti-rabies PEP.

In our study, 8.5% of the victims did not receive timely PEP treatment. The WHO advises that immediate washing and flushing of wounds with soap and water for at least 15 minutes, or water alone, and disinfection with substances with anti-viral activity is essential after exposure to rabies virus (20). Taking immediate care of all bites and scratches is necessary and essential because the virus can remain within the site of the injury for an indefinite period (21). Joseph et.al reported a high proportion (41%) of the study subjects did not receive prompt PEP (22). A report by an anti-rabies clinic in a government hospital showed that 73.5% of the animal bite victims completed the course of intramuscular rabies vaccination. The report mentioned the key reasons of not completing the vaccination as loss of wages, forgotten dates, costs incurred, and distance from the hospital (23). Likewise, another analysis from urban slums of Chennai, in India among 301 participants also showed that the compliance with intra‑dermal rabies vaccination was just 55.1%; the complaint ones offered reasons such as with noncompliance loss of wages and interference with school timings (24). Some respondents mistakenly thought that the incubation period was so long that prompt PEP was not needed. In fact, immediate PEP is important for neutralize of the rabies virus in bite site to prevent its spread into the central nervous system (25). In Khazaei et al.'s study, in Khalilabad county of Khorasan Razavi province, Iran, 93.4% of the victims received PEP in less than 48 h of exposure (26). Certain critical factors (e.g., infection site, and severity, substantial delays in the initiation, improper or incomplete care of the wound, lack or inappropriate administration of rabies immunoglobulin, and the overall biological quality) were associated with prophylaxis failures investigated (27).

The present study found a higher rate of delay among women compared to men victims of animal bite (11.4% compared to 7.8%). A study conducted in shiraz province, Iran (28), which had similar results to our study, showed that delay of more than 48 h in PEP, was higher in women. And in studies conducted in northeastern Iran, there was no significant difference between PEP in terms of gender, place of residence and damaged organ (8, 10).

As demonstrated in a previous study, some people may think that domestic animals are less dangerous than roaming ones (29). On the contrary, 99% of cases of human rabies are related to domestic dogs (30). This finding may demonstrate some degree of self- risk assessment, where bite victims do not complete the treatment course when they believe the risk is small.

On the presence of watchdogs in most rural households in the study area, appropriate education programs should be provided for the teaching of behavioral skills in high-risk groups. Similarly, those who live far from health centers, and are in lower socioeconomic classes undergo longer delays in receiving PEP, which increases the risk of developing rabies (8).

In our study, delay more than 48 h belonged to the rustic region. Because of development of the primary health care (PHC) in Iran in past decades, and availability of health houses and health centers in almost all villages, it seems that there is no physical barrier to access the care animal bite victims (31). It is reported in Tanzania that a distance of more than 10 kilometers from vaccination centers is a significant contributor in PEP delay (17). In Narlidere District, in Turkey, in rural areas the vaccination rate for post-exposure rabies vaccination was higher than urban area (32). People living in remote areas of developing countries have difficulty accessing public health services (33).

## Conclusion

Based on various variables that affect the initiation of PEPbeing bitten by a carnivorous, being bitten in the rural places, and especially being a female. Development of educational programs for dog owners, especially in rural areas, and increasing public awareness may help us prevent the delay in initiation of PEP for animal bite victims. 

## Limitation

First, given the limitations of the cross-sectional study design, it is not possible to claim causal effect. Due to the study’s retrospective design, authors were unable to collect other variables such as education level, past history of rabies vaccination, socioeconomic status, and other variables related to delay in receiving PEP. Possible recall bias by the animal bite victims in remembering the time of bite may result in information bias in calculation of delay of treatments.

## Funding

This study was supported by the Tabriz University of Medical sciences, Tabriz, Iran. And the fund number was 60198.
